# Sidt2 inhibits islet β-cell dedifferentiation by regulating insulin secretion

**DOI:** 10.1016/j.jbc.2025.110544

**Published:** 2025-07-30

**Authors:** Jing Gu, Meng-Xiang Qi, Rui-Xi Zhang, Ying-Ying Song, Xing Hu, Hai-Jun Liu, Ya-Ting Zhang, Wen-Xiu Wu, Ya-Jun Wu, Jia-Hao Xu, Jun-Hao Wang, Jing-Rong Li, Miao-Miao Liu, Wen-Jun Pei, Yao Zhang, Li-Zhuo Wang, Jia-Lin Gao

**Affiliations:** 1 Department of Endocrinology and Genetic Metabolism, The First Affiliated Hospital of Wannan Medical College (Yijishan Hospital of Wannan Medical College), Wuhu, PR China; 2 Institute of Endocrine and Metabolic Diseases, Department of Endocrinology and Genetic Metabolism, The First Affiliated Hospital of Wannan Medical College (Yijishan Hospital of Wannan Medical College), Wuhu, PR China; 3 Department of Endocrinology, Kunshan Fourth People's Hospital, Kunshan, PR China; 4 Anhui Province Key Laboratory of Biological Macro-molecules Research, Wannan Medical College, Wuhu, PR China; 5 Department of Biochemistry and Molecular Biology, Wannan Medical Collage, Wuhu, PR China; 6 School of Clinical Medicine, Wannan Medical College, Wuhu, PR China; 7 Key Laboratory of Non-coding RNA Transformation Research of Anhui Higher Education Institution, Wannan Medical College, Wuhu, PR China

**Keywords:** diabetes, islet β-cell dedifferentiation, islet β-cell identity, insulin secretion, Sidt2

## Abstract

β-cell dedifferentiation plays an important role in the pathogenesis of type 2 diabetes mellitus (T2DM). SID1 transmembrane family member 2 (Sidt2) is a lysosomal membrane protein known to regulate hepatic steatosis and lipid metabolism. However, its role in pancreatic β-cell dedifferentiation remains unclear. In this study, we found that Sidt2 expression was significantly decreased in diabetic mice and patients, correlating with impaired glucose metabolism. Through *in-vitro* and *in-vivo* experiments, we observed that the loss of Sidt2 accelerated β-cell dedifferentiation, as evidenced by an increase in the number of α cells and a marked reduction in key β-cell markers, such as pancreatic and duodenal homeobox 1 (Pdx1), V-maf musculoaponeurotic fibrosarcoma oncogene homolog A (MafA), and glucose transporter 2 (Glut2). Moreover, Sidt2 deficiency disrupted islet function, leading to impaired insulin secretion. Further analyses revealed that the dedifferentiation of β cells induced by Sidt2 deficiency was independent of the Forkhead box protein O1 (FoxO1) pathway, a known regulator of β-cell identity. Instead, the primary mechanism appeared to be related to defects in insulin secretion. In conclusion, our study identified a novel regulatory mechanism of β-cell dedifferentiation and insulin secretion mediated by Sidt2. These findings enhance our understanding of the molecular mechanisms underlying β-cell dedifferentiation and offer new perspectives on the pathogenesis of T2DM, supporting the potential of targeting Sidt2 as an innovative therapeutic strategy to preserve β-cell function and to treat this disease.

Type 2 diabetes mellitus (T2DM) accounts for more than 90% of all diabetic cases and has long been a major global health challenge (1). The rapidly rising incidence of T2DM is alarming, with the number of affected individuals expected to rise to 300 million by 2025 and 643 million by 2030 (2). These projections underscore the urgent need for novel prevention and treatment strategies to address the growing prevalence of T2DM worldwide. The pathogenesis of T2DM is primarily characterized by two interrelated events: insulin resistance, which involves reduced glucose uptake and increased glucose production, and islet β-cell failure, manifested by impaired β-cell function and enhanced α-cell activity (3). β-cell dedifferentiation is a critical mechanism driving β-cell failure, featured by the loss of β-cell identity and their transformation into nonfunctional, progenitor-like cells (4). Growing evidence has supported reversing β-cell dedifferentiation and promoting β-cell regeneration as potential strategies for treating T2DM (5).

Insulin secretory granules (ISGs) are single-layer membrane organelles that are essential for the packaging, storage, and secretion of insulin (6). These granules contain insulin-related peptides, prohormone convertases, and various vesicle membrane proteins, all of which are crucial for regulating glucose metabolism (7). Lysosomes, with their single-layer membrane structure, exhibit significant similarity to ISGs, suggesting that lysosome membrane proteins (LMPs) may be involved in the formation, maturation, and exocytosis of ISGs (8). Accumulating studies have highlighted the involvement of LMPs in glucose metabolism-related processes, including glucose transmembrane transport, glycolysis, and glucose metabolism. For instance, vesicle-associated membrane protein (Vamp) 7, an LMP associated with membrane transport, plays a key role in regulating autophagy to maintain the mitochondrial mass and to mediate insulin secretion from islet β cells (9). Similarly, lysosome-associated membrane protein-3 has been shown to protect nascent ISGs from degradation, thereby preventing β-cell failure and sustaining insulin secretion in T2DM (10). These findings highlight the significant link between LMPs and glucose metabolism in the pathogenesis of T2DM.

SID1 transmembrane family member 2 (Sidt2) is an LMP that is closely associated with glucose metabolism (11). Systemic knockout (KO) of *Sidt2* in mice led to abnormal glucose metabolism, which was characterized by elevated blood sugar levels and impaired glucose tolerance (12). Moreover, abnormal blood glucose levels in *Sidt2*^−/−^ mice have been attributed to altered calcium release from ISGs (13). In addition, our preliminary data revealed a significant decrease in serum Sidt2 levels in patients with T2DM. Given that β-cell dedifferentiation is a critical mechanism in the pathogenesis of T2DM, we hypothesized that Sidt2 might be involved in this process.

Therefore, this study aimed to explore the relationship between Sidt2 and β-cell dedifferentiation by elucidating how Sidt2 affects β-cell function, as well as to uncover its potential implications for the management of T2DM.

## Results

### Sidt2 expression was low in diabetic mice and closely related to glucose metabolism

**Figure 1:**
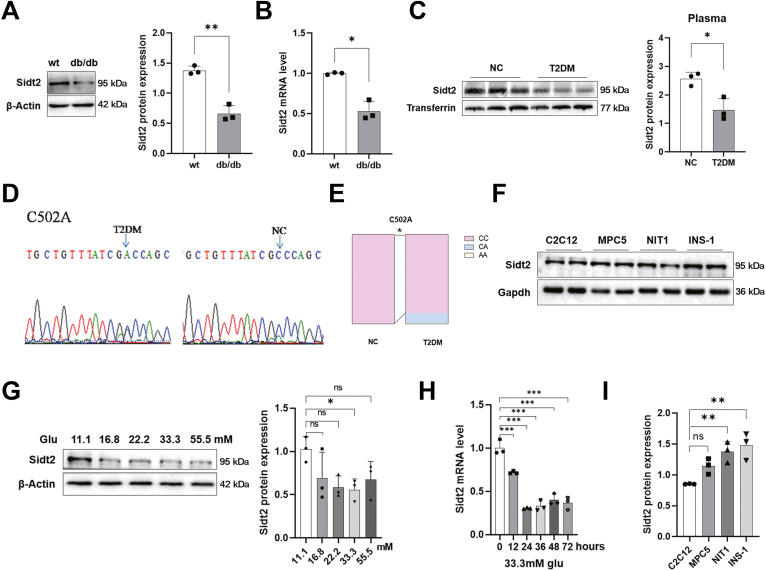
**Sidt2 expression was low in diabetics and related to glucose metabolism.** (*A* and *B*) Sidt2 protein and mRNA levels in the islet cells of WT and diabetic db/db mice (12 weeks old). (*C*) Plasma Sidt2 protein expression in healthy individuals and T2DM patients. (*D*) C502A SNP diagram of the human *Sidt2* promoter. (*E*) Variation ratio of the C502A SNP in healthy individuals (*n* = 100) and T2DM patients (*n* = 100). (*F*) Sidt2 protein expression in different cell lines. (*G*) Sidt2 protein expression in INS-1 cells after 24 h of stimulation with different concentrations of glucose. (*H*) *Sidt2* mRNA levels in INS-1 cells after stimulation with 33.3 mM glucose for 0, 12, 24, 36, 48, and 72 h. (*I*) Statistical analysis of data from *panel* (*F*). ∗*p* < 0.05, ∗∗*p* < 0.01, ∗∗∗*p* < 0.001.

To explore the relationship between Sidt2 and diabetes, we first examined Sidt2 expression in the islet cells of diabetic db/db mice and found a significant reduction compared to that in wild-type (WT) mice (Fig. 1, *A* and *B*). Similarly, the plasma levels of Sidt2 were significantly lower in diabetic patients than in healthy individuals (Fig. 1*C*). Sequencing analysis of the *Sidt2* promoter in 100 clinical samples revealed that the SNP C502A was associated with T2DM (Fig. 1*D*). The incidence of C502A in T2DM patients was 12%, which is significantly greater than that found in healthy controls (Fig. 1*E*), indicating a vital role of *Sidt2* in T2DM. Further *in vitro* analysis showed that Sidt2 was highly expressed in the islet cell lines NIT1 and INS-1 (Fig. 1, *F* and *I*). Notably, Sidt2 expression was closely affected by the glucose level. Western blot analysis showed that the protein levels of Sidt2 were decreased with an increasing glucose concentration, with the most significant inhibition observed at 33.3 mM (Fig. 1*G*). qRT-PCR analysis confirmed that the *Sidt2* mRNA levels declined with prolonged glucose exposure (33.3 mM) in INS-1 cells (Fig. 1*H*), indicating that Sidt2 expression was negatively regulated by glucotoxicity. Moreover, using *Sidt2* KO mice, we found that compared with WT mice, *Sidt2* KO mice exhibited significantly impaired glucose tolerance and insulin tolerance as well as elevated fasting blood glucose levels at an early stage (Fig. S1, *A*–*E*), while their body weight was reduced (Fig. S1*F*). All of these findings were consistent with the characteristics of T2DM. Collectively, Sidt2 expression was low in diabetes and closely related to glucose metabolism.

### *Sidt2* KO resulted in an increase in glucagon expression and the α/β cell ratio, inhibiting the proliferation of islet β cells

**Figure 2:**
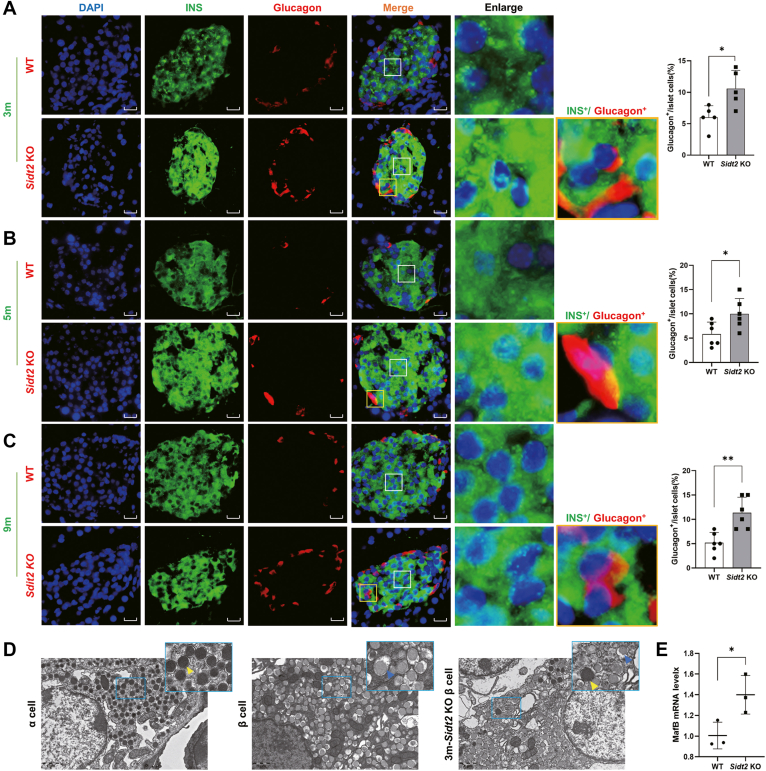
**The α/β cell ratio was increased in *Sidt2* KO mice.** (*A*) Immunofluorescence staining of insulin (*green*) and glucagon (*red*) in 3-month-old mice. Scale bar = 20 μm. (*B*) Immunofluorescence staining of insulin (*green*) and glucagon (*red*) in the pancreas of 5-month-old mice. Scale bar = 20 μm. (*C*) Immunofluorescence staining of insulin (*green*) and glucagon (*red*) in the pancreas of 9-month-old mice. Scale bar = 20 μm. (*D*) Transmission electron microscopy images showing the location of insulin and glucagon-like particles in *Sidt2* KO islet cells. The particle properties are indicated by colored arrows: glucagon (*yellow*), insulin (*blue*). Scale bar = 1 μm. (*E*) *MafB* mRNA levels in *Sidt2* KO islets. ∗*p* < 0.05, ∗∗*p* < 0.01.

Dedifferentiation plays an important role in islet β-cell dysfunction, which is often manifested as the loss of the mature β-cell phenotype. The transdifferentiation of β cells into α cells, leading to an increased α/β cell ratio, is a key indicator of dedifferentiation (14). In this study, immunofluorescence double staining was used to detect the expression of insulin and glucagon in mice at 1, 3, 5, and 9 months of age. The results showed no significant change in glucagon expression in 1-month-old mice (Fig. S2*A*). However, at 3 months, *Sidt2* KO mice began to show increased glucagon expression, with the presence of double hormone-positive cells (Glu^+/^INS^+^) (Fig. 2*A*). The number of Glu^+^ cells remained significantly greater in *Sidt2* KO mice than in WT mice at both 5 months (Fig. 2*B*) and 9 months (Fig. 2*C*) of age. The presence of Glu^+/^INS^+^ cells was also detected in the *Sidt2* KO group.

Glucagon granules in islet α cells typically display a crescent-shaped cap-like gap between the membrane and the core (15). Transmission electron microscopy detected glucagon granules in the islet β cells of 3-month-old *Sidt2* KO mice, further confirming the existence of Glu^+/^INS^+^ cells (Fig. 2*D*). V-maf musculoaponeurotic fibrosarcoma oncogene homolog B (MafB) is an islet α-cell marker that is only expressed in islet α cells after adulthood (16). The transcription levels of *MafB* in islet cells were elevated in 3-month-old *Sidt2* KO mice compared to the WT group (Fig. 2*E*). Additionally, *Sidt2* KO affected the survival of islet β cells. Flow cytometry and the CCK-8 assay demonstrated that Sidt2 promoted β-cell apoptosis and inhibited cell proliferation (Fig. S3, *A* and *B*). Consistently, western blot analysis showed that the protein expression of cleaved caspase 3, cleaved caspase 9, cleaved poly ADP-ribose polymerase, and Bax was significantly increased after *Sidt2* KO, while Bcl-2 expression was notably reduced, indicating enhanced apoptosis of islet β cells in the *Sidt2* KO group (Fig. S3, *C*–*F*). In summary, *Sidt2* KO resulted in increased glucagon expression, a higher α/β cell ratio, and inhibition of β-cell proliferation in mice.

### The β-cell dedifferentiation marker Ngn3 was upregulated in *Sidt2* KO mice

**Figure 3:**
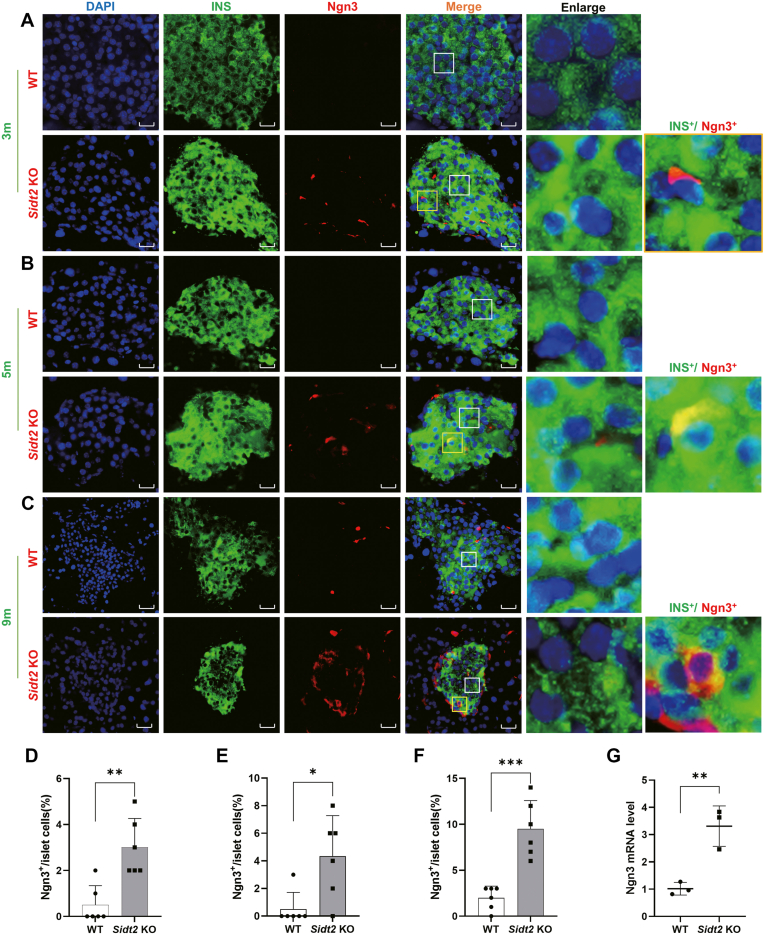
**Increased expression of β-cell dedifferentiation markers in *Sidt2* KO mice.** (*A*) Immunofluorescence staining of insulin (*green*) and Ngn3 (*red*) in the pancreas of 3-month-old mice. Scale bar = 20 μm. (*B*) Immunofluorescence staining of insulin (*green*) and Ngn3 (*red*) in the pancreas of 5-month-old mice. Scale bar = 20 μm. (*C*) Immunofluorescence staining of insulin (*green*) and Ngn3 (*red*) in the pancreas of 9-month-old mice. Scale bar = 20 μm. (*D*) Statistical analysis of data from *panel* (*A*). (*E*) Statistical analysis of data from *panel* (*B*). (*F*) Statistical analysis of data from *panel* (*C*). (*G*) *Ngn3* mRNA levels in WT and *Sidt2* KO mice. ∗*p* < 0.05, ∗∗*p* < 0.01, ∗∗∗*p* < 0.001.

Ngn3 is a specific marker of islet progenitor cells, and its increased expression often indicates β-cell dedifferentiation (17). Immunofluorescence double staining of insulin and Ngn3 was then performed to analyze the expression and distribution of Ngn3 in the islets of mice at 1, 3, 5, and 9 months of age. At 1 month, insulin was abundantly expressed in both WT and *Sidt2* KO mice, while Ngn3 was weakly expressed and nearly undetectable in both groups, with no significant difference observed between the two groups (Fig. S2*B*). At 3 months, Ngn3 expression remained rarely detected in WT mice, similar to the results of 1-month-old mice, whereas the *Sidt2* KO mice exhibited a significant increase in Ngn3 expression (Fig. 3*A*). Statistical analysis revealed a marked increase in the number of Ngn3^+^ cells per unit islet area in *Sidt2* KO mice (Fig. 3*D*). In addition, some cells that co-expressed insulin and Ngn3 (Ngn3^+^/INS^+^) were also observed in *Sidt2* KO mice (Fig. 3*A*). The trend of increased Ngn3 expression persisted in the 5-month-old *Sidt2* KO mice (Fig. 3*E*), with a significant rise in Ngn3^+^/INS^+^ cells compared to the WT mice (Fig. 3*B*). The expression differences in Ngn3 continued at 9 months of age (Fig. 3*C*), with *Sidt2* KO mice exhibiting higher proportions of both Ngn3^+^ cells and Ngn3^+^/INS^+^ (Fig. 3, *C* and *F*). Additionally, transcriptional analysis confirmed that the *Ngn3* mRNA levels were significantly elevated in the *Sidt2* KO mice (Fig. 3*G*), consistent with the immunofluorescence findings. Thus, the upregulation of Ngn3 suggests that β-cell dedifferentiation was occurring in *Sidt2* KO mice.

### Sidt2 ablation induced the loss of β-cell identity markers both *in vitro* and *in vivo*

**Figure 4:**
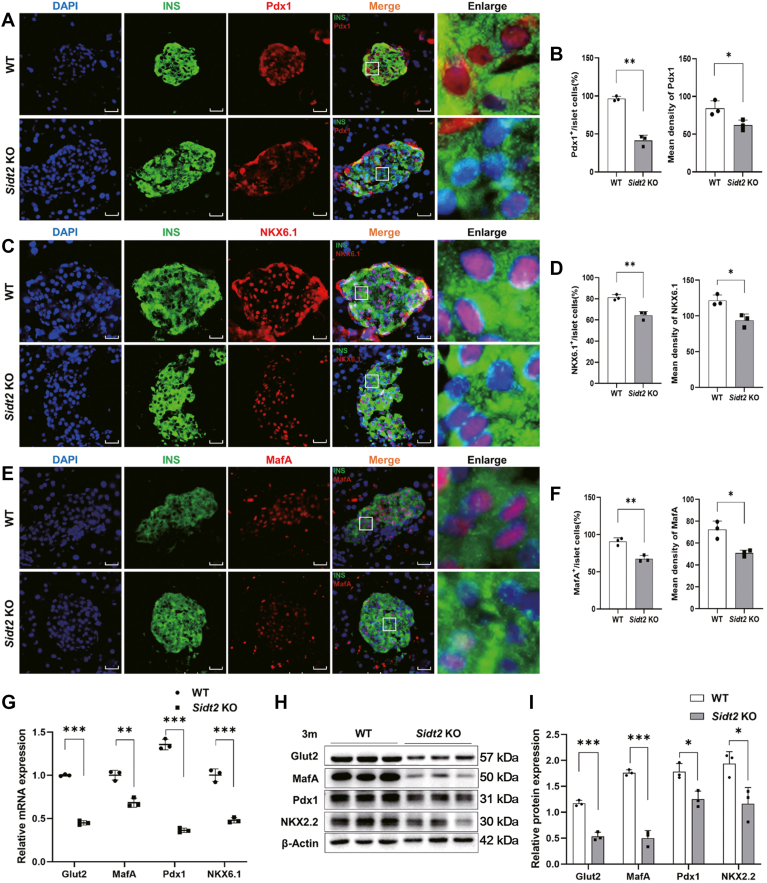
**Sidt2 ablation induced the loss of β-cell identity markers *in vivo.*** (*A*) Immunofluorescence staining of insulin (*green*) and Pdx1 (*red*) in the pancreas of 3-month-old mice. Scale bar = 20 μm. (*B*) Statistical analysis of data from *panel* (*A*). (*C*) Immunofluorescence staining of insulin (*green*) and NKX6.1 (*red*) in the pancreas of 3-month-old mice. Scale bar = 20 μm. (*D*) Statistical analysis of data from *panel* (*C*). (*E*) Immunofluorescence staining of insulin (*green*) and MafA (*red*) in the pancreas of 3-month-old mice. Scale bar = 20 μm. (*F*) Statistical analysis of data from *panel* (*E*). (*G*) The mRNA levels of β-cell markers in WT and *Sidt2* KO mice. (*H*) The protein expression levels of β-cell markers in WT and *Sidt2* KO mice. (*I*) Statistical analysis of data from *panel* (*H*). ∗*p* < 0.05, ∗∗*p* < 0.01, ∗∗∗*p* < 0.001.

A significant decline in β-cell identity markers is another important indicator of β-cell dedifferentiation (18). Key mature β-cell markers include pancreatic and duodenal homeobox 1 (Pdx1), NK6 homeobox 1 (NKX6.1), NK2 homeobox 2 (NKX2.2), V-maf musculoaponeurotic fibrosarcoma oncogene homolog A (MafA), and glucose transporter 2 (Glut2). To assess the expression of these markers, we performed immunofluorescence staining, qRT-PCR, and western blot assays. Immunofluorescence detection revealed that Pdx1^+/^INS^+^ cells in the *Sidt2* KO group represented only 41.3% of the total INS^+^ cells per unit area, which was significantly less compared to the 95% observed in the WT group (Fig. 4, *A* and *B*). Similarly, NKX6.1^+/^INS^+^ cells in the *Sidt2* KO group accounted for 64.3% of the islet INS^+^ cells per unit area, which was also significantly less than the 81.3% seen in the WT group (Fig. 4, *C* and *D*). Correspondingly, the proportion of MafA^+^/INS^+^ cells showed a marked decrease in the *Sidt2* KO group, with only 67.5% compared to 90.7% in the WT group (Fig. 4, *E* and *F*). Further analyses of the transcription and translation levels also confirmed that the mRNA levels of the islet β-cell markers Glut2, MafA, Pdx1, and NKX6.1 were significantly reduced in *Sidt2* KO mice (Fig. 4, *G*–*I*), aligning with the immunofluorescence staining results.

**Figure 5:**
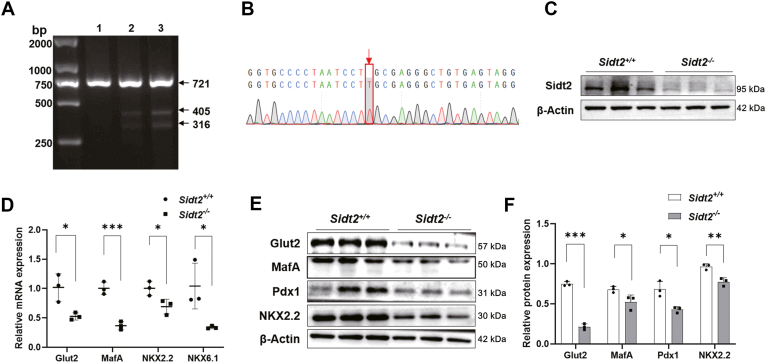
**Sidt2 ablation induced the loss of β-cell identity markers *in vitro.*** (*A*) T7E1 enzyme validation of *Sidt2*^−/−^ INS-1 cells constructed using CRISPR-Cas9 technology (1: *Sidt2*^+/+^ INS-1 cells; 2–3: *Sidt2*^−/−^ INS-1 cells). (*B*) Sequencing map of *Sidt2*^−/−^ INS-1 monoclonal cells. (*C*) Verification of Sidt2 protein levels in *Sidt2*^+/+^ and *Sidt2*^−/−^ INS-1 cells. (*D*) mRNA levels of β-cell markers in *Sidt2*^+/+^ and *Sidt2*^−/−^ INS-1 cells. (*E*) Protein expression of β-cell markers in *Sidt2*^+/+^ and *Sidt2*^−/−^ INS-1 cells. (*F*) Statistical analysis of data from *panel* (*E*). ∗*p* < 0.05, ∗∗*p* < 0.01, ∗∗∗*p* < 0.001.

In addition to *in vivo* studies, we performed *in vitro* verification at the cellular level. A Sidt2-deleted INS-1 monoclonal cell line (*Sidt2*^−/−^ INS-1) was constructed using CRISPR-Cas9 technology. The deletion was confirmed through T7E1 enzyme validation, Sanger sequencing, and western blot analysis (Fig. 5, *A*–*C*). Subsequently, qRT-PCR and western blot assays were performed to measure the expression levels of islet β-cell markers, including Glut2, MafA, NKX2.2, and NKX6.1. As expected, Sidt2 KO significantly reduced the expression of islet β-cell markers at both the mRNA and protein levels (Fig. 5, *D*–*F*). The above results collectively suggest that the absence of Sidt2 led to the loss of β-cell identity and triggered β-cell dedifferentiation.

### Endogenous Sidt2 was partially colocalized with Vamp2 and insulin in the vesicles of ISGs in β cells

**Figure 6:**
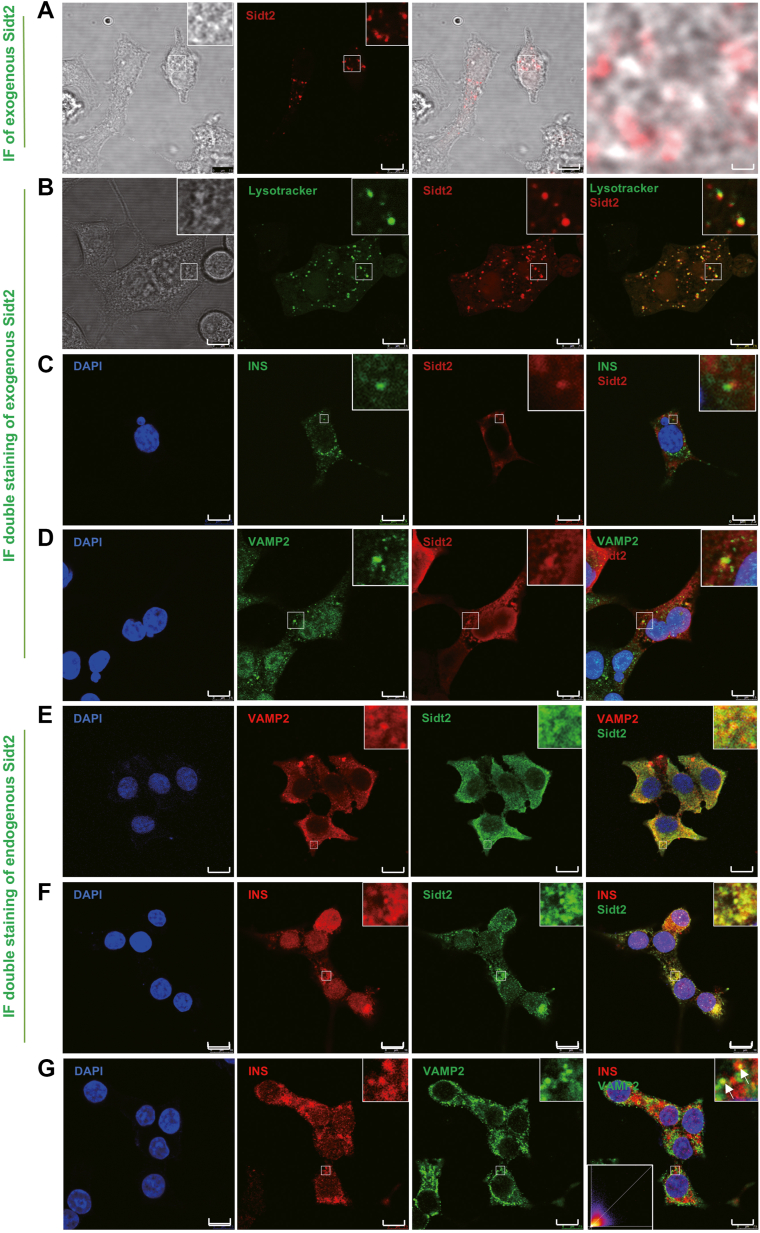
**The localization of Sidt2 in INS-1 cells.** (*A*) Immunofluorescence staining of overexpressed Sidt2 (mCherry) in INS-1 cells. (*B*) Immunofluorescence double staining of overexpressed Sidt2 (mCherry) and Lysotracker (*green*) in INS-1 cells. (*C*) Immunofluorescent double staining of overexpressed Sidt2 (mCherry) and Vamp2 (*green*) in INS-1 cells. (*D*) Immunofluorescence double staining of overexpressed Sidt2 (mCherry) and insulin (*green*) in INS-1 cells. (*E*) Immunofluorescence double staining of endogenous Sidt2 (*red*) and Vamp2 (*green*) in INS-1 cells. (*F*) Immunofluorescence double staining of endogenous Sidt2 (*green*) and insulin (*red*) in INS-1 cells. (*G*) Immunofluorescence double staining of endogenous Vamp2 (*green*) and insulin (*red*) in INS-1 cells. Scale bar = 10 μm.

Fusion protein expression vectors, which allow for the construction of fusion proteins with specific tags, are widely used in the identification of protein subcellular localization owing to their specificity and noninterfering characteristics (19). To investigate the localization of Sidt2 in β cells, we constructed a vector containing exogenous Sidt2 labeled with mCherry and transfected it into INS-1 cells. The results revealed that the mCherry fluorescence was predominantly localized in the cytoplasm (Fig. 6*A*). Lysotracker Green, a lysosomal tag used to label acidic organelles, including lysosomes and insulin granules, was further used to investigate the location of Sidt2. Analysis of the fluorescence from Lysotracker Green and mCherry demonstrated that Sidt2 was primarily distributed in acidic vesicles, indicating a high degree of colocalization between Sidt2 and acidic organelles (Fig. 6*B*). Vamp2, an LMP located on the vesicles of ISGs, is a classic marker of ISGs (20). Immunofluorescence double staining of Sidt2 and Vamp2 revealed colocalization in INS-1 cells (Fig. 6*C*). Additionally, co-staining with an insulin antibody exhibited that Sidt2 was partially colocalized with insulin (Fig. 6*D*). These results suggest that exogenous Sidt2 was partially distributed on the membrane of acidic vesicles, such as ISGs. To further investigate the subcellular distribution of endogenous Sidt2 in islet β cells, we performed immunofluorescence double staining using antibodies against Sidt2, Vamp2, and insulin. These results showed that endogenous Sidt2 was partially colocalized with both Vamp2 and insulin (Fig. 6, *E* and *F*). The Pearson correlation coefficient between Vamp2 and insulin was 0.65 (Fig. 6*G*), indicating a moderately strong positive linear relationship. Collectively, the above findings imply that Sidt2 might be a new ISG membrane protein.

### Sidt2 depletion led to insulin secretion disorder and intracellular insulin retention in mice

**Figure 7:**
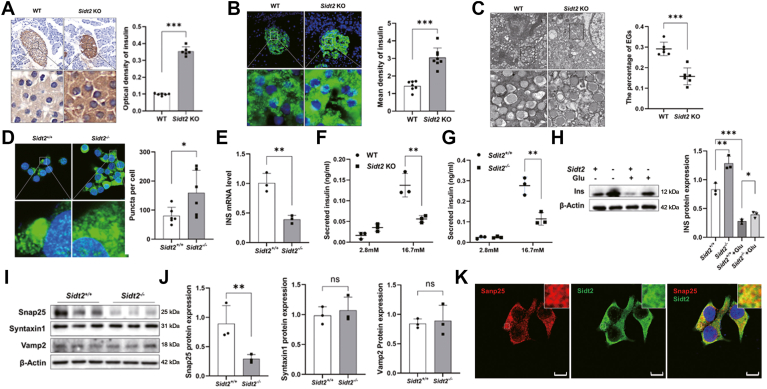
**Insulin secretion disorder after Sidt2 KO.** (*A*) Immunohistochemical staining of insulin in the pancreatic tissues of 3-month-old mice. The optical density of insulin was measured. Scale bar = 20 μm. (*B*) Immunofluorescence staining of insulin in the pancreatic tissues of 3-month-old mice. The average fluorescence intensity was calculated. Scale bar = 20 μm. (*C*) Ultrastructural morphology of islet β cells in WT and *Sidt2* KO mice. The proportion of empty granules was calculated. Scale bar = 1 μm. (*D*) Immunofluorescence staining of insulin in *Sidt2*^+/+^ and *Sidt2*^−/−^ INS-1 cells. The fluorescence puncta were measured. Scale bar = 10 μm. (*E*) mRNA expression levels of insulin in *Sidt2*^+/+^ and *Sidt2*^−/−^ INS-1 cells. (*F*) GSIS assay of primary islets in WT and *Sidt2* KO mice. (*G*) GSIS assay of *Sidt2*^*+/+*^ and *Sidt2*^*−/−*^ INS-1 cells. (*H*) Western blot analysis of the intracellular insulin protein content following high-glucose stimulation (16.7 mM). (*I*) Protein expression levels of the SNARE complex in INS-1 cells. (*J*) Statistical analysis of data from *panel* (*I*). (*K*) Immunofluorescence double staining of endogenous Sidt2 (*green*) and Snap25 (*red*) in INS-1 cells. ∗*p* < 0.05, ∗∗*p* < 0.01, ∗∗∗*p* < 0.001.

Given that Sidt2 has been verified to colocalize with insulin, we further explored whether Sidt2 is correlated with the insulin secretion of islet β cells. At the tissue level, both immunohistochemistry and immunofluorescence analyses revealed a significant increase in the insulin content in *Sidt2* KO mice (Fig. 7, *A* and *B*). Transmission electron microscopy showed an expansion of the endoplasmic reticulum and a decrease in the number of empty insulin granules in *Sidt2* KO mice (Fig. 7*C*). Additionally, *Sidt2*^−/−^ INS-1 cells displayed a significant increase in insulin granules compared to *Sidt2*^+/+^ INS-1 cells (Fig. 7*D*). Interestingly, despite the increased insulin granules, the mRNA level of insulin in *Sidt2*^−/−^ INS-1 cells was reduced (Fig. 7*E*), indicating that Sidt2 deletion did not enhance insulin production.

To further explore the underlying cause of the increase in the intracellular insulin content, a GSIS test was performed. The results showed that the insulin secretion capacity was significantly decreased in both primary islets from *Sidt2* KO mice (Fig. 7*F*) and in *Sidt2*^−/−^ INS-1 cells (Fig. 7*G*), suggesting a disruption in the exocytosis and secretion of insulin granules following *Sidt2* deletion. Additional analysis revealed that the intracellular insulin content was significantly increased in *Sidt2*^−/−^ cells compared to *Sidt2*^+/+^ cells. Moreover, glucose stimulation led to a significant reduction in the insulin content in both the Sidt2^+/+^ and *Sidt2*^−/−^ groups compared to the unstimulated controls (Fig. 7*H*). We also examined the expression of key proteins involved in ISG exocytosis and found that synaptosome-associated protein 25 (Snap25) expression was significantly reduced in the *Sidt2*^−/−^ group, while the levels of syntaxin one and Vamp2 showed no significant difference (Fig. 7, *I* and *J*). Immunofluorescence double-staining experiments revealed the colocalization of Sidt2 and Snap25 in INS-1 cells (Fig. 7*K*). These data suggest that Sidt2 depletion disrupted insulin secretion and exocytosis, leading to intracellular insulin retention.

### Islet β-cell dedifferentiation after Sidt2 ablation may be attributed to dysregulated insulin secretion and is independent of the Forkhead box protein O1 (FoxO1) pathway

**Figure 8:**
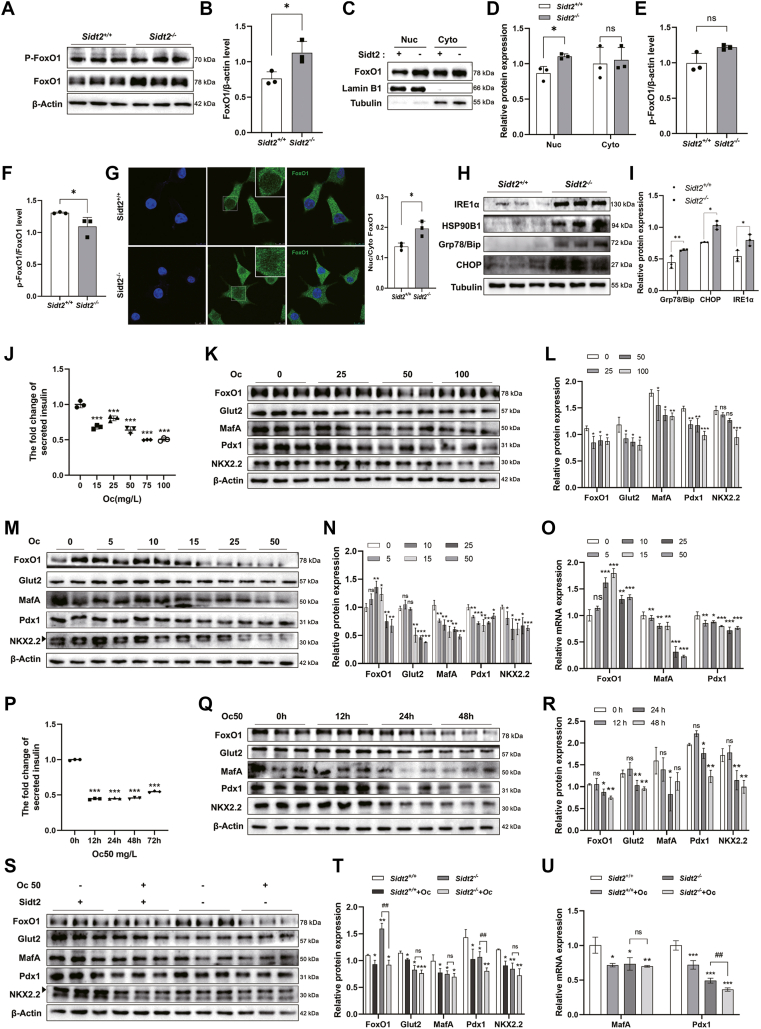
**Disrupted insulin secretion induced β-cell dedifferentiation after Sidt2 ablation.** (*A*) Protein expression of FoxO1 and p-FoxO1 in *Sidt2*^+/+^ and *Sidt2*^−/−^ INS-1 cells. (*B*, *E* and *F*) Statistical analysis of data from *pane*l (*A*). (*C*) Protein expression of FoxO1 after separation of nuclear and cytoplasmic fractions in *Sidt2*^+/+^ and *Sidt2*^−/−^ INS-1 cells. (*D*) Statistical analysis of data from *panel* (*C*). (*G*) Immunofluorescence staining of FoxO1 in *Sidt2*^+/+^ and *Sidt2*^−/−^ INS-1 cells. Scale bar = 10 μm. (*H*) Protein expression of IRE1α, HSP90B1, Grp78/Bip, and CHOP in *Sidt2*^+/+^ and *Sidt2*^−/−^ INS-1 cells. (*I*) Statistical analysis of data from *panel* (*H*). (*J*) Insulin content in the supernatant of INS-1 cells stimulated with octreotide (Oc) at different concentrations. (*K*) Protein expression of β-cell markers in INS-1 cells stimulated with Oc at different concentrations for 24 h. (*L*) Statistical analysis of data from panel (*K*). (*M*) Protein expression of β-cell markers in INS-1 cells stimulated with Oc at 0 to 50 mg/L. (*N*) Statistical analysis of data from *panel* (*M*). (*O*) mRNA levels of FoxO1, MafA, and Pdx1 in INS-1 cells stimulated with Oc at 0 to 50 mg/L. (*P*) Insulin content in the supernatant of INS-1 cells stimulated with 50 mg/L Oc at different times. (*Q*) Protein expression of β-cell markers in INS-1 cells stimulated with 50 mg/L Oc at different times. (*R*) Statistical analysis of data from panel (*Q*). (*S*) Protein expression of β-cell markers in in *Sidt2*^*+/+*^ and *Sidt2*^*−/−*^ INS-1 cells stimulated with 50 mg/L Oc for 24 h. (*T*) Statistical analysis of data from panel (*S*). (*U*) mRNA levels of MafA and Pdx1 in *Sidt2*^*+/+*^ and *Sidt2*^*−/−*^ INS-1 cells stimulated with 50 mg/L Oc for 24 h. ∗*p* < 0.05, ∗∗*p* < 0.01, ∗∗∗*p* < 0.001, ^##^*p* < 0.01. *n* = 3.

The dedifferentiation of islet β cells is closely related to the expression of FoxO1 (21). To investigate whether β-cell dedifferentiation depends on the classic FoxO1 regulatory pathway, we analyzed FoxO1 expression and its phosphorylation state in *Sidt2*^−/−^ INS-1 cells. Our results showed an increase in the total FoxO1 protein levels in *Sidt2*^−/−^ INS-1 cells. The total FoxO1 protein was composed of phosphorylated FoxO1 (p-FoxO1), which resides in the cytoplasm, and dephosphorylated FoxO1, which translocates to the nucleus (Fig. 8, *A*–*D*). Further analysis revealed a decreased p-FoxO1/FoxO1 ratio in *Sidt2*^−/−^ INS-1 cells compared to *Sidt2*^+/+^ INS-1 cells (Fig. 8*D*), suggesting that there is relatively more dephosphorylated FoxO1 in the nucleus. This finding implies that the increase in FoxO1 expression observed in *Sidt2*^−/−^ INS-1 cells may be a compensatory response rather than a direct cause of dedifferentiation. Nucleoplasm separation experiments supported this by showing enhanced nuclear expression in *Sidt2*^−/−^ INS-1 cells than in *Sidt2*^+/+^ INS-1 cells (Fig. 8, *E* and *F*). Additionally, immunofluorescence staining showed an increased nuclear/cytoplasmic ratio of FoxO1 expression in *Sidt2*^−/−^ INS-1 cells (Fig. 8*G*), confirming a compensatory increase in nuclear FoxO1 in the absence of Sidt2. These results collectively suggest that islet β-cell dedifferentiation induced by Sidt2 deficiency occurred independently of the FoxO1 pathway. Moreover, Sidt2^−/−^ induced a pronounced endoplasmic reticulum (ER) stress response, as evidenced by the upregulation of key molecular chaperones (Grp78/Bip and HSP90B1), the ER stress sensor (IRE1α), and the proapoptotic factor CHOP (Fig. 8, *H* and *I*).

To delve further into the relationships between Sidt2 deficiency and insulin secretion, insulin secretion disorder, and β-cell dedifferentiation, we established an *in vitro* model using octreotide (Oc), which is an inhibitor of insulin secretion, in INS-1 cells (Fig. 8, *J* and *P*). Interestingly, high doses of Oc (25, 50, and 100 mg/L) significantly decreased the expression levels of FoxO1 and β-cell markers (Fig. 8, *K* and *L*). However, when the Oc concentration was reduced, a notable increase in FoxO1 expression was observed, especially with 15 mg/L Oc, mirroring the compensatory FoxO1 expression increase seen in *Sidt2*^−/−^ INS-1 cells (Fig. 8, *M*–*O*). Moreover, prolonged Oc treatment led to a significant reduction in key β-cell markers, including Glut2, MafA, Pdx1, and NKX2.2 (Fig. 8, *Q* and *R*), indicating that Oc induced β-cell dedifferentiation by inhibiting insulin secretion and that continuous suppression of insulin secretion might affect β-cell identity and further exacerbate dedifferentiation. Interestingly, when *Sidt2*^−/−^ INS-1 cells were treated with 50 mg/L Oc for 24 h, there were no significant changes in the expression levels of Glut2, MafA, and NKX2.2 (Fig. 7, *S*–*U*), suggesting that Sidt2 KO might play a similar role as Oc. In summary, the dedifferentiation of β cells following Sidt2 ablation may be a consequence of insulin secretion dysfunction.

### GM partially restored *Sidt2* KO-induced β-cell dedifferentiation

**Figure 9:**
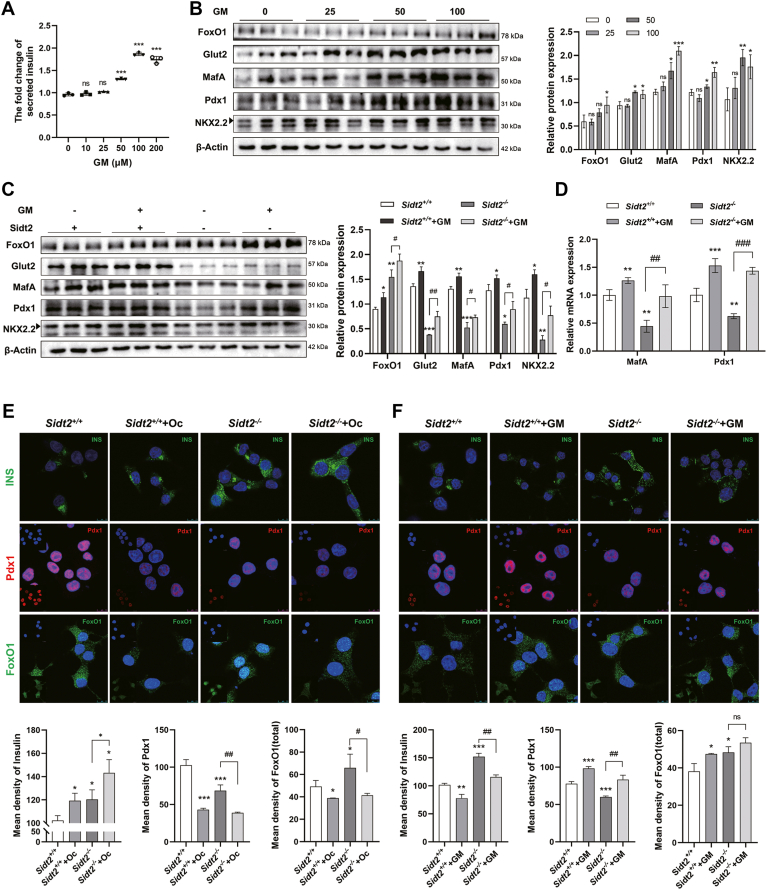
**Promoting insulin secretion partially mitigated β-cell dedifferentiation disorder induced by *Sidt2* KO.** (*A*) Insulin content in the supernatant of INS-1 cells stimulated with different concentrations of glimepiride (GM). (*B*) Protein expression of β-cell markers in INS-1 cells stimulated with different concentrations of GM. (*C*) Protein expression of β-cell markers in *Sidt2*^*+/+*^ and *Sidt2*^*−/−*^ INS-1 cells stimulated with 100 μM GM (*D*) mRNA levels of MafA and Pdx1 in *Sidt2*^*+/+*^ and *Sidt2*^*−/−*^ INS-1 cells stimulated with 100 μM GM. (*E*) Immunofluorescence staining of INS, Pdx1, and FoxO1 in *Sidt2*^*+/+*^ and *Sidt2*^*−/−*^ INS-1 cells stimulated with 50 mg/L octreotide. Scale bar = 10 μm. (*F*) Immunofluorescence staining of INS, Pdx1, and FoxO1 in *Sidt2*^*+/+*^ and *Sidt2*^*−/−*^ INS-1 cells stimulated with 100 μM GM. Scale bar = 10 μm. ∗*p* < 0.05, ∗∗*p* < 0.01, ∗∗∗*p* < 0.001; ^#^*p* < 0.05, ^##^*p* < 0.01, ^###^*p* < 0.001. *n* = 3.

**Figure 10:**
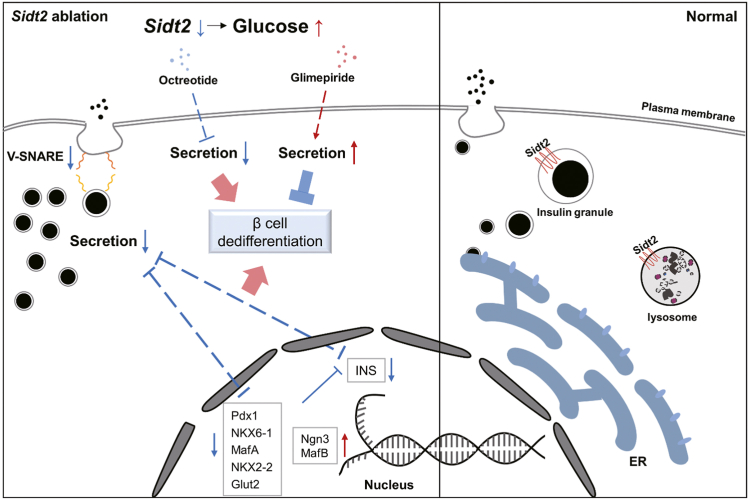
**Effect and regulation of Sidt2 on islet β-cell differentiation.** Sidt2 interacts with blood glucose. *Sidt2* deficiency inhibits insulin secretion and accelerates β-cell dedifferentiation.

GM is a drug used in the treatment of T2DM that promotes insulin secretion. To further explore the relationship between insulin secretion and β-cell dedifferentiation, we treated INS-1 cells with GM at different concentrations for 1 h. Insulin secretion significantly increased with higher GM concentrations, plateauing at 100 μM (Fig. 9*A*). Concurrently, the expression levels of β-cell markers, such as Glut2, MafA, Pdx1, and NKX2.2, were also elevated with increasing GM concentrations, even at 100 μM GM (Fig. 9*B*). These findings indicate that GM not only enhanced insulin secretion but also maintained the differentiation status of islet β cells. Furthermore, when GM (100 μM) was administered to *Sidt2*^−/−^ INS-1 cells, there was a partial restoration in the expression of Glut2, MafA, Pdx1, and NKX2.2 (Fig. 9, *C* and *D*). Immunofluorescence staining showed that the insulin content in the *Sidt2*^−/−^ + Oc group was significantly increased compared to the *Sidt2*^−/−^ group, while Pdx1 expression and the mean density of FoxO1 expression were decreased, with levels similar to those in the *Sidt2*^+/+^ + Oc group (Fig. 9*E*). Compared to the *Sidt2*^−/−^ group, the intracellular insulin content in the *Sidt2*^−/−^ + GM group was decreased, while Pdx1 expression was partially restored (Fig. 9*F*). These findings indicate that GM stimulation partially corrected insulin secretion disorder and restored the upregulation of β-cell markers that are lost due to Sidt2 deficiently. Taken together, Sidt2 deletion-induced disturbance in insulin secretion plays a key role in β-cell dedifferentiation. The ability of GM to partially reverse these effects highlights the potential therapeutic approach of targeting insulin secretion to prevent or mitigate β-cell dedifferentiation in T2DM. The proposed mechanism is shown in Figure 10.

## Discussion

Regulating insulin secretion and β-cell dedifferentiation is crucial in the management and progression of T2DM, as these processes play pivotal roles in maintaining glucose homeostasis and preserving the function of insulin-producing β cells (22). In this study, we showed that Sidt2 was significantly downregulated in diabetic patients and mice, and it was closely related to glucose metabolism. In addition, *Sidt2* KO enhanced β-cell dedifferentiation, aggravated islet dysfunction, and inhibited insulin secretion from β cells. Moreover, dysregulated insulin secretion, but not the FoxO1 pathway, may be responsible for *Sidt2* KO-induced β-cell dedifferentiation.

ISGs serve as the storage and transport vessels for insulin (23), yet studies of ISG membrane proteins are limited. To date, only a few such proteins, such as Vamp2, Vamp3, and syntaxin 6, have been identified (20). They play crucial roles in controlling the movement, secretion, release, and degradation of ISGs (6). For instance, Vamp2 mediates the fusion of predocked ISGs, forming a “primed” or fusion-ready complex (24). The dysfunction or absence of these proteins, however, can potentially lead to diabetic phenotypes (25). Our previous study has demonstrated that Sidt2 is widely expressed in various tissues, such as the liver, kidney, pancreas, and skeletal muscle, and *Sidt2* KO decreased the insulin secretion ability in mice (11). In this study, we observed abnormal Sidt2 expression in both diabetic patients and mice. In addition, *Sidt2* KO mice developed a diabetic phenotype, which was characterized by increased glucose and insulin tolerance as well as elevated fasting blood glucose levels. Further investigation revealed that endogenous Sidt2 colocalized with Vamp2 and insulin, suggesting that Sidt2 is not only an LMP but also present in ISG membranes within β cells. The soluble *N*-ethylmaleimide-sensitive factor attachment protein receptor (SNARE) complex is essential for anchoring vesicles to the plasma membrane during ISG exocytosis and for the release of insulin from vesicle granules (26). In the current study, we observed significant downregulation of Snap25, a core protein of the SNARE complex, in Sidt2^−/−^ INS-1 cells compared to Sidt2^+/+^ INS-1 cells, highlighting the critical role of Sidt2 in insulin exocytosis. Lysosomes and ISGs, both single-layer vesicle-like structures, share similar biogenesis and formation processes, including endoplasmic reticulum synthesis, Golgi processing, and vesicle secretion (26). This strong homology in production and processing may underlie the dual biological function of Sidt2.

β-cell dedifferentiation is a key factor in the onset and development of T2DM, wherein terminally differentiated islet β cells lose their specialized functions (27). Although β cells are terminally differentiated, their differentiation process is not irreversible (28). Increasing evidence has indicated that mature β cells, under certain conditions, can partially or fully lose their differentiated phenotype and cellular identity, regressing to a less differentiated or precursor-like state (29). This phenomenon was observed in our study, where *Sidt2* KO led to an increase in dedifferentiated β cells. Dedifferentiation is often accompanied by an increase in α cells, likely due to the reprogramming of β cells into α cells (30), as evidenced by an increased α/β cell ratio observed after *Sidt2* KO. The primary function of mature islet β cells is to synthesize and secrete insulin, which is crucial for maintaining blood glucose homeostasis (31). Key proteins that are involved in glucose metabolism in β cells and respond to glucose stimulation are essential for insulin secretion and release (32). In this study, cells coexpressing insulin and glucagon were found in the islets of adult *Sidt2* KO mice and persisted over time, indicating β-cell dedifferentiation. Numerous glucagon granules were also detected in some β cells of *Sidt2* KO mice. β-cell dedifferentiation is also marked by the loss of key β-cell transcription factors, such as Pdx1, MafA, and NKX6.1 (33). Conversely, genes typically expressed in pancreatic progenitor cells, such as *Ngn3*, were upregulated in dedifferentiated β cells (34). In this study, we observed a significant reduction in the expression of Pdx1, MafA, and NKX6.1 in 3-month-old Sidt2 KO mice compared to WT controls. Concurrently, INS^+^/Ngn3^+^ cells gradually emerged in the islets. These findings support that *Sidt2* KO induced β-cell dedifferentiation.

The FoxO1 pathway is a well-established regulatory mechanism in β-cell dedifferentiation (35). FoxO1 translocates to the nucleus in response to high glucose concentrations or other metabolic stresses, enhancing the expression of β-cell markers such as Pdx1 and MafA as well as resulting in augmented insulin synthesis and secretion necessary for β-cell function (21). However, persistent hyperglycemia can lead to reduced FoxO1 expression, compromising β-cell identity and function, thereby contributing to β-cell dedifferentiation (36). In the present study, we found that the increased nuclear FoxO1 expression was a compensatory response to metabolic stress, contrasting with the typical dedifferentiation observed when the FoxO1 levels were downregulated. Furthermore, our findings suggest that β-cell dedifferentiation induced by *Sidt2* KO occurred independently of the FoxO1 pathway, indicating that Sidt2 may influence β-cell identity through alternative mechanisms. A previous study had shown that both absolute and relative insulin deficiencies can significantly impact the differentiation status of islet β cells, with restoration of insulin expression partially reversing β-cell dedifferentiation (37). The United Kingdom Prospective Diabetes Study has reported that β-cell function usually declines at an average rate of approximately 4% with disease progression, though patients treated with sulfonylureas retained better β-cell function compared to those on metformin or a prescribed diet alone (38). In this study, we used the insulin inhibitor Oc to construct an insulin secretion disorder model *in vitro* and found that the FoxO1 levels initially increased but then decreased as the Oc concentration rose from 0 mg/L to 50 mg/L. This change was accompanied by diminished β-cell identity, as evidenced by the decreased expression of key β-cell markers (*e.g.*, Glut2, MafA, Pdx1, and NKX2.2), which was consistent with the dedifferentiation observed in the *Sidt2* KO mice. GM can promote insulin secretion and is used clinically to manage T2DM (39). Additionally, Brereton *et al.* have reported that mutations in the ATP-sensitive potassium channel, which impair insulin exocytosis, are associated with β-cell dedifferentiation in mice and that GM is more effective than insulin at reversing this dedifferentiation and controlling hyperglycemia (40). Our study showed that while Oc inhibited insulin secretion and weakened β-cell identity in Sidt2^−/−^ INS-1 cells, the addition of GM improved insulin secretion and partially restored β-cell identity. These findings collectively suggest a novel regulatory mechanism where Sidt2 KO induced insulin secretion disorders and mediated β-cell dedifferentiation, with potential therapeutic implications for managing β-cell dysfunction in T2DM.

This study has several limitations that need to be addressed. First, current technology constraints prevent real-time observation of the dynamic interactions between Sidt2 and ISGs. Consequently, it remains uncertain whether Sidt2 interacts with ISGs through specific binding sites or directly acts on the ISG membrane. Additionally, while the relationship between insulin secretion and β-cell dedifferentiation was demonstrated using pharmacological agents such as GM and Oc in cell culture experiments, these findings have not yet been validated in animal models. To fully elucidate the underlying mechanisms and to assess the therapeutic potential of targeting Sidt2 for preserving β-cell function, further *in vivo* studies are necessary.

In conclusion, our study revealed a novel regulatory role of Sidt2 in both β-cell dedifferentiation and insulin secretion. The identification of Sidt2 as a potential regulator of insulin secretion provides new insights for developing targeted strategies in the prevention and treatment of T2DM.

## Experimental procedures

### Mass spectrometric analysis of human plasma samples

Blood samples (5 ml each) were extracted from three healthy individuals and placed into anticoagulant tubes. The samples were then centrifuged at 3000 rpm for 10 min to separate the plasma from blood cells. The plasma was subsequently delivered to BiotechPack company for protein identification. A secondary peptide sequence map and predictions of protein content were obtained. The study protocol was approved by the Medical Ethics Committee of Yijishan Hospital (Approval number: LLSC-2018-012) and was performed in compliance with the Declaration of Helsinki. All participants provided written informed consent.

### Whole blood DNA extraction and single-nucleotide polymorphism (SNP) identification

A 5-mL sample of venous blood was collected from fasting individuals and, following sufficient anticoagulation, stored at −80 °C. Genomic DNA was extracted from the blood using a Whole Blood Genomic Extraction Kit (DP304, Tiangen), according to the manufacturer’s instructions. The *Sidt2* promoter was amplified using PCR with the following primers (F: 5′-TAAGGGCAAATGCGGGACC-3′; R: 5′-TGAGAAGGAGGGCGGCTG-3′). The C502A variant, which had a length of 324 bp, was directly sequenced using an Applied Biosystems 3730 automatic sequencer (The Beijing Genomics Institute).

### Cell culture

INS-1 cells (RRID: CVCL_0352) were purchased from the Shanghai Institute of Biochemistry and Cell Biology, Chinese Academy of Sciences. They were cultured in RPMI-1640 medium supplemented with 10% fetal bovine serum and 50 μM β-mercaptoethanol (Gibco; Invitrogen), and incubated at 37 °C with 5% CO_2_. All cell culture experiments were conducted using INS-1 cells between passage 5 and 20.

### *Sidt2* KO using CRISPR/Cas9

LentiCRISPR-V2 plasmids (RRID: Addgene_52961) were used as the vector for this experiment. The sgRNA sequences (F: 5′-CAGGTGCCCCTAATCCTGCG-3′, R: 5′-CGCAGGATTAGGGGCACCTG-3′) were designed using the online tool available at http://chopchop.cbu.uib.no/. The plasmids were linearized using the BsmBI enzyme (E1602, New England Biolabs), and the sgRNA sequences were inserted into the plasmids using T4 ligase (M0202; New England Biolabs) to construct the lenticRISPR-V2-SIDT2-sgRNA plasmids. 293T cells were transfected with LenticRISPR-V2 and LenticRISPR-v2-SIDT2-sgRNA plasmids using a second-generation lentivirus packaging system to produce high-titer viruses. The collected lentiviruses were then transfected into INS-1 cells, and puromycin was used to screen resistant cells. The T7E1 enzyme (E3321, New England Biolabs) was used to confirm the presence of mutated bases. Monoclonal cells were subsequently selected to construct stable *Sidt2*^+/+^ and *Sidt2*^−/−^ INS-1 monoclonal cell lines. The DNA sequencing was performed using the following primers (F: 5′-TCCAAGGGCGAGCTTCTTCA-3′, R: 5′-GGGCTATACAGTGCCTCCTAT-3′), and the sequencing was completed by The Beijing Genomics Institute.

### Cloning of *Sidt2*

The cDNA of *Sidt2* was cloned into the HBAD-Adeasy-mCherry vector using the Adeasy Adenovirus Packaging System (Hanbio Biotechnology Co. Ltd), allowing simultaneous expression of *Sidt2* and mCherry. INS-1 cells were infected with the virus at a multiplicity of infection of 300, and the virus titer was above 10^10^ plaque-forming units/ml. After confirming the successful construction of the model *via* qRT-PCR and western blot to detect *Sidt2* expression, INS-1 cells overexpressing Sidt2 were incubated in a working solution of 50 nM Lysotracker Green (40738ES50, Yeasen) at 37 °C for 1 h. The solution was then removed, and fresh culture medium was added. The staining was observed under a Leica microscope.

### Octreotide (Oc) and glimepiride (GM) treatments

INS-1 cells were cultured in RPMI 1640 medium with 0 to 100 mg/L octreotide acetate (Hy-17365, MedChemExpress) for 0 to 48 h. The supernatant was collected, and the insulin content was measured using an insulin enzyme-linked immunosorbent assay (ELISA) kit (E-EL-R2466C, Elabscience Biotechnology Inc) to determine the optimal concentration and incubation time for inhibiting insulin secretion by Oc.

For GM treatment, INS-1 cells were balanced in glucose-free Krebs–Ringer bicarbonate buffer (KRBH) solution for 1 h, followed by incubation in 16 mM glucose KRBH solution containing 0 to 200 μM GM (HY-B0104, MedChemExpress) for 1 h. The insulin content in the supernatant was then measured using an insulin ELISA kit to determine the optimal GM concentration for stimulating insulin secretion.

### Fluorescein isothiocyanate (FITC)/propidium iodide (PI) double staining

Apoptosis was detected using a FITC/PI kit (556547, BD Biosciences, Franklin Lakes, NJ, USA). Cells were first digested with 0.25% trypsin, then resuspended in cold phosphate-buffered saline (PBS), and centrifuged at 1000 rpm for 5 min. The collected cells were resuspended twice in cold PBS and centrifuged at 1000 rpm for 5 min. Approximately 1 × 10 (6) cells/ml were resuspended in 1 × Binding Buffer. A 100-μl aliquot of this suspension (1 × 10 (5) cells) was transferred to an Eppendorf tube. Then, 5 μl of FITC and 5 μl of PI were added to the tube, which was gently vortexed and incubated in the dark for 15 min. Finally, 400 μl of 1 × Binding Buffer was added to each tube, and flow cytometry was performed following a 1-h incubation.

### Cell viability detection

The cell counting kit-8 (CCK-8) assay (BS350A, BioSharp) was used to detect cell viability. Cells were seeded in 96-well plates and cultured for 0 to 48 h. At different time points, 10 μl of CCK-8 solution was added to each well, and the reaction was continued for 1 h. The absorbance at 450 nm was measured using a microplate reader, and the optical density value at 0 h was used for normalization. CCK-8 curves were plotted at different time points to reflect cell viability.

### Separation of nuclear and cytoplasmic proteins

Cells were collected and resuspended in 100 μl of Buffer A (P0028; Beyotime). The suspension was incubated on ice for 15 min and then centrifuged at 12,000 rpm and 4 °C for 1 min. The pellet was washed with 1 ml of Buffer A three times and resuspended with 150 μl of Buffer B. After centrifugation at 12,000 rpm and 4 °C for 30 min, the supernatant containing cytoplasmic proteins was removed. The protein concentration was detected using the bicinchoninic acid method (P0009; Beyotime). The subsequent steps were performed in accordance with the standard western blot procedure.

### Animals

Twelve-week-old WT and diabetic db/db mice (B6 background) were purchased from the Shanghai Model Organisms Center, Inc. Diabetic db/db mice (B6 background) were obtained from Shanghai Model Organisms Center, Inc. *Sidt2* KO mice were generated using Cre-LoxP gene targeting technology, as described previously (41). The construction of these mice was carried out by the Shanghai Southern Model Company. All animal experiments were approved by the Animal Ethics Committee of Wannan Medical College (Approval number: LLSC-2018-012).

### Glucose tolerance test

After fasting for 12 h, the mice were administered with 2 g/kg of D-glucose solution *via* intraperitoneal injection. Blood samples were collected from the tail tips, and the blood glucose levels were detected at 0, 15, 30, 60, 90, and 120 min using a glucose meter (Sinocare CA-3).

### Insulin tolerance test

After fasting for 6 h, the mice were administered with 0.75 U/kg of insulin solution *via* intraperitoneal injection. Blood samples were collected from the tail tips, and the blood glucose levels were measured at 0, 15, 30, 45, 60, 90, and 120 min using a glucose meter (Sinocare CA-3).

### Immunohistochemistry staining

Three to five mice from each group were randomly selected and sacrificed by cervical dislocation. Their pancreases were harvested and fixed in 4% paraformaldehyde. Paraffin-embedded slices with a thickness of 5 μm were prepared, dewaxed, and stained following antigen retrieval. Images were captured using an Olympus microscope, and the average optical density was calculated using ImageJ software.

### Transmission electron microscopy

Transmission electron microscopy was performed on pancreatic tissue specimens from three WT and three *Sidt2* KO male mice. The specimens were prepared according to the same protocol as mentioned previously. After identifying the islet under a light microscope, sections of the pancreas were examined, with one of six consecutive sections selected. A minimum of 10 slices was taken from each mouse. Random images were captured, and 2 to 3 images with a magnification of 20,000 × were selected for each mouse. The ultrastructural changes between the WT and *Sidt2* KO groups were observed, and the ratio of the number of empty granules to the total number of insulin secretion granules was calculated.

### Isolation and purification of islets

Mice were sacrificed by cervical dislocation. A 32-G needle was inserted into the duodenal papilla, and the common bile duct was retrogradely injected with 1 mg/ml collagenase V solution (C9263, Sigma) until the pancreas was fully inflated. The excised pancreas was digested at 37 °C for 28 min, followed by vortexing for 10 s. To stop digestion, 15 ml of cold Hanks’ balanced salt solution was added, and the mixture was centrifuged 1000 rpm and 4 °C for 2 min. The supernatant was discarded, and the washing process was repeated. Islets were isolated using Histopaque-1077 (10771, Sigma) and manually selected under a microscope. To extract islet proteins, 1 μl of radioimmunoprecipitation assay buffer was added to each islet. Approximately 100 to 120 islets could be purified from a single mouse.

### Glucose-stimulated insulin secretion (GSIS) test

The purified islets were incubated overnight in RPMI 1640 medium with 10% fetal bovine serum to restore islet function. A 12-well plate was prepared, with 20 islets placed in each well to ensure that the size and number of islets in each well were consistent. KRBH at a volume of 750 μl was added to each well. After a 1-h incubation at 37 °C, the islets were transferred to a new 12-well plate containing KRBH buffer plus 2.8 mM glucose and incubated at 37 °C for another hour. After incubation, 100 μl of supernatant was collected into an Eppendorf tube, representing the basal insulin secretion level. The islets were then transferred to a new 12-well plate with KRBH buffer plus 16.7 mM glucose and incubated at 37 °C for 1 h. Another 100 μl of supernatant was collected into an Eppendorf tube, which contained insulin stimulated with glucose. The supernatant was centrifuged and stored at −80 °C. The insulin levels in the supernatant were determined using an insulin ELISA kit (EZRMI, Millipore).

### Immunofluorescence staining

Cells were plated uniformly on confocal dishes and fixed in 4% paraformaldehyde for 10 min when the cell density was appropriate. Then, the cells were washed three times with PBS and permeabilized in PBS solution containing 5% bovine serum albumin and 0.3% Triton X-100 at room temperature for 1 h. After an overnight incubation with primary antibodies at 4 °C, the cells were washed three times with PBS and incubated with a secondary antibody at room temperature for 90 min. The cells were then stained with 4′,6-diamidino-2-phenylindole (DAPI) for 10 min, washed three times with PBS, and photographed.

Pancreatic specimens were collected from 3 to 5 mice per group after perfusion, sectioned into 5-μm frozen sections, and fixed with 4% paraformaldehyde. The tissue sections were then blocked, stained with primary antibodies at 4 °C overnight, and then incubated with a fluorescent secondary antibody at room temperature for 90 min. After washing with PBS, DAPI was used for counterstaining, and the sections were photographed.

For the double-immunofluorescence staining, the samples were incubated with primary antibodies from different species (mouse/rabbit) simultaneously. Secondary antibodies conjugated with green/red fluorescent probes (Alex Fluor 488/594) corresponding to the primary antibodies (goat anti-mouse/rabbit) were also incubated simultaneously. The subsequent steps followed the same protocol as the immunofluorescence staining experiment. The antibodies used in this experiment are listed in Table S1.

All images were captured using Leica and Olympus microscopes. At least three mice were used for each group, with a minimum of six pancreatic slices per mouse. Each slice contained no fewer than 30 islets, and the total number of islet cells was more than 1000. The average fluorescence intensity and fluorescence puncta were quantified using ImageJ software. To determine the proportion of positive cells, ImageJ software was used to count the total number of INS^+^ cells as well as glucagon^+^/neurogenin3 (Ngn3)^+^ cells and other transcription factor-positive cells in the islets. The proportion of positive cells between the WT and *Sidt2* KO mice was compared.

### RNA extraction and quantitative reverse transcription polymerase chain reaction (qRT-PCR)

RNA was extracted using the Trizol method and reverse transcribed using RevertAid RT (K1691, Thermo Fisher Scientific) as previously described (42). The sequences of the primers used in this experiment are shown in Tables S2 and S3.

### Western blot

Western blot assays were performed according to a previously published protocol (12). Each experiment was repeated independently at least three times. Beta-actin was used as an internal control to normalize the gray values, which were quantified using ImageJ software. The antibodies used in this experiment are shown in Table S1.

### Statistical analysis

Data are expressed as the means ± standard error of the mean. Differences between the two groups were assessed using an unpaired *t* test. Statistical analysis was conducted using Graphics 9.0 software, with *p* < 0.05 considered statistically significant.

## Data availability

All data needed to evaluate the conclusions in the paper are present in the paper and/or the Supplementary Materials.

## Supporting information

This article contains supporting information.

## Conflict of interest

The authors declare that they have no conflicts of interest related to the content of this study.

## References

[1] Dunlay S.M., Givertz M.M., Aguilar D., Allen L.A., Chan M., Desai A.S. (2019). Type 2 diabetes mellitus and heart failure: a scientific statement from the American heart association and the heart failure society of America: this statement does not represent an update of the 2017 ACC/AHA/HFSA heart failure guideline update. Circulation.

[2] Chandrasekaran P., Weiskirchen R. (2024). The role of obesity in type 2 diabetes Mellitus-An overview. Int. J. Mol. Sci..

[3] Javeed N., Matveyenko A.V. (2018). Circadian etiology of type 2 diabetes mellitus. Physiology (Bethesda).

[4] Son J., Accili D. (2023). Reversing pancreatic β-cell dedifferentiation in the treatment of type 2 diabetes. Exp. Mol. Med..

[5] Ghasemi Gojani E., Rai S., Norouzkhani F., Shujat S., Wang B., Li D. (2024). Targeting β-Cell plasticity: a promising approach for diabetes treatment. Curr. Issues Mol. Biol..

[6] Norris N., Yau B., Kebede M.A. (2021). Isolation and proteomics of the insulin secretory granule. Metabolites.

[7] Germanos M., Gao A., Taper M., Yau B., Kebede M.A. (2021). Inside the insulin secretory granule. Metabolites.

[8] Gu J., Geng M., Qi M., Wang L., Zhang Y., Gao J. (2021). The role of lysosomal membrane proteins in glucose and lipid metabolism. FASEB J..

[9] Aoyagi K., Ohara-Imaizumi M., Itakura M., Torii S., Akimoto Y., Nishiwaki C. (2016). VAMP7 regulates autophagy to maintain mitochondrial homeostasis and to control insulin secretion in pancreatic β-Cells. Diabetes.

[10] Pasquier A., Vivot K., Erbs E., Spiegelhalter C., Zhang Z., Aubert V. (2019). Lysosomal degradation of newly formed insulin granules contributes to β cell failure in diabetes. Nat. Commun..

[11] Gao J., Yu C., Xiong Q., Zhang Y., Wang L. (2015). Lysosomal integral membrane protein Sidt2 plays a vital role in insulin secretion. Int. J. Clin. Exp. Pathol..

[12] Gao J., Gu X., Mahuran D.J., Wang Z., Zhang H. (2013). Impaired glucose tolerance in a mouse model of sidt2 deficiency. PLoS One.

[13] Islam M.S. (2010). Calcium signaling in the islets. Adv. Exp. Med. Biol..

[14] Saleh M., Gittes G.K., Prasadan K. (2021). Alpha-to-beta cell trans-differentiation for treatment of diabetes. Biochem. Soc. Trans..

[15] Yin Q., Ni Q., Wang Y., Zhang H., Li W., Nie A. (2020). Raptor determines beta-cell identity and plasticity independent of hyperglycemia in mice. Nat. Commun..

[16] Russell R., Carnese P.P., Hennings T.G., Walker E.M., Russ H.A., Liu J.S. (2020). Loss of the transcription factor MAFB limits β-cell derivation from human PSCs. Nat. Commun..

[17] Cheng C.W., Villani V., Buono R., Wei M., Kumar S., Yilmaz O.H. (2017). Fasting-mimicking diet promotes Ngn3-Driven beta-cell regeneration to reverse diabetes. Cell.

[18] Talchai C., Xuan S., Lin H.V., Sussel L., Accili D. (2012). Pancreatic beta cell dedifferentiation as a mechanism of diabetic beta cell failure. Cell.

[19] Nishimura W., Takahashi S., Yasuda K. (2015). MafA is critical for maintenance of the mature beta cell phenotype in mice. Diabetologia.

[20] Guest P.C. (2019). Biogenesis of the insulin secretory granule in health and disease. Adv. Exp. Med. Biol..

[21] Casteels T., Zhang Y., Frogne T., Sturtzel C., Lardeau C.H., Sen I. (2021). An inhibitor-mediated beta-cell dedifferentiation model reveals distinct roles for FoxO1 in glucagon repression and insulin maturation. Mol. Metab..

[22] Rorsman P., Ashcroft F.M. (2018). Pancreatic β-Cell electrical activity and insulin secretion: of mice and men. Physiol. Rev..

[23] Vakilian M., Tahamtani Y., Ghaedi K. (2019). A review on insulin trafficking and exocytosis. Gene.

[24] Aslamy A., Thurmond D.C. (2017). Exocytosis proteins as novel targets for diabetes prevention and/or remediation?. Am. J. Physiol. Regul. Integr. Comp. Physiol..

[25] Zhu D., Zhang Y., Lam P.P., Dolai S., Liu Y., Cai E.P. (2012). Dual role of VAMP8 in regulating insulin exocytosis and islet β cell growth. Cell Metab..

[26] Omar-Hmeadi M., Idevall-Hagren O. (2021). Insulin granule biogenesis and exocytosis. Cell Mol. Life Sci..

[27] Efrat S. (2019). Beta-cell dedifferentiation in type 2 diabetes: concise review. Stem Cells.

[28] Bensellam M., Jonas J.C., Laybutt D.R. (2018). Mechanisms of β-cell dedifferentiation in diabetes: recent findings and future research directions. J. Endocrinol..

[29] Nair G.G., Liu J.S., Russ H.A., Tran S., Saxton M.S., Chen R. (2019). Recapitulating endocrine cell clustering in culture promotes maturation of human stem-cell-derived β cells. Nat. Cell Biol..

[30] Yin Q., Ni Q., Wang Y., Zhang H., Li W., Nie A. (2020). Raptor determines β-cell identity and plasticity independent of hyperglycemia in mice. Nat. Commun..

[31] Campbell J.E., Newgard C.B. (2021). Mechanisms controlling pancreatic islet cell function in insulin secretion. Nat. Rev. Mol. Cell Biol..

[32] Merrins M.J., Corkey B.E., Kibbey R.G., Prentki M. (2022). Metabolic cycles and signals for insulin secretion. Cell Metab..

[33] Zhang M., Yang C., Zhu M., Qian L., Luo Y., Cheng H. (2021). Saturated fatty acids entrap PDX1 in stress granules and impede islet beta cell function. Diabetologia.

[34] Swisa A., Avrahami D., Eden N., Zhang J., Feleke E., Dahan T. (2017). PAX6 maintains β cell identity by repressing genes of alternative islet cell types. J. Clin. Invest..

[35] Talchai C., Xuan S., Lin H.V., Sussel L., Accili D. (2012). Pancreatic β cell dedifferentiation as a mechanism of diabetic β cell failure. Cell.

[36] Kitamura T. (2013). The role of FOXO1 in β-cell failure and type 2 diabetes mellitus. Nat. Rev. Endocrinol..

[37] Szabat M., Page M.M., Panzhinskiy E., Skovsø S., Mojibian M., Fernandez-Tajes J. (2016). Reduced insulin production relieves endoplasmic reticulum stress and induces β cell proliferation. Cell Metab..

[38] King P., Peacock I., Donnelly R. (1999). The UK prospective diabetes study (UKPDS): clinical and therapeutic implications for type 2 diabetes. Br. J. Clin. Pharmacol..

[39] Muller-Wieland D., Kellerer M., Cypryk K., Skripova D., Rohwedder K., Johnsson E. (2018). Efficacy and safety of dapagliflozin or dapagliflozin plus saxagliptin versus glimepiride as add-on to metformin in patients with type 2 diabetes. Diabetes Obes. Metab..

[40] Brereton M.F., Iberl M., Shimomura K., Zhang Q., Adriaenssens A.E., Proks P. (2014). Reversible changes in pancreatic islet structure and function produced by elevated blood glucose. Nat. Commun..

[41] Geng M.Y., Wang L., Song Y.Y., Gu J., Hu X., Yuan C. (2021). Sidt2 is a key protein in the autophagy-lysosomal degradation pathway and is essential for the maintenance of kidney structure and filtration function. Cell Death Dis..

[42] Hu Y., Wang Y., Zhang X., Jin X., Pei W., Wang L. (2020). TM7SF1, an important autophagy regulatory protein in mouse podocytes. Biochem. biophysical Res. Commun..

