# Supplementary material for: Sidt2 inhibits islet β-cell dedifferentiation by regulating insulin secretion
Source: J Biol Chem. 2025 Jul 30;301(9):110544. doi: 10.1016/j.jbc.2025.110544 (PMC12406273; doi:10.1016/j.jbc.2025.110544)
Supplement: Supporting Figures and Tables [file mmc1.docx]

**Table S1.** Antibodies

| Antibody | Use | Dilution | Source |
| --- | --- | --- | --- |
| Mouse anti-β-Actin | WB | 1:5000 | Sigma-Aldrich A5316 |
| Rabbit anti-GAPDH | WB | 1:1000 | CST 5174 |
| Rabbit anti-Sidt2 | WB | 1:1000 | Abnova PAB27211 |
| Rabbit anti-Insulin | WB/IF | 1:1000/1:200 | CST 3014 |
| Mouse anti-Insulin | IF | 1:500 | CST 8138 |
| Mouse anti-Snap25 | WB | 1:200 | Santa Cruz sc-376713 |
| Mouse anti-Syntaxin1 | WB | 1:200 | Santa Cruz sc-12736 |
| Mouse anti-Vamp2 | WB/IF | 1:200 | Santa Cruz sc-20039 |
| Rabbit anti-MafA | WB | 1:1000 | ABclonal A18662 |
| Rabbit anti-MafA | IF | 1:100 | Abcam ab264418 |
| Rabbit anti-Pdx1 | WB | 1:1000 | ABclonal A10173 |
| Mouse anti-Pdx1 | IF | 1:100 | DSHB F109-D12 |
| Rabbit anti-NKX2.2 | WB | 1:1000 | ABclonal A16696 |
| Rabbit anti-Glut2 | WB | 1:1000 | ABclonal A12307 |
| Rabbit anti-Glucagon | IF | 1:200 | CST 2760 |
| Rabbit anti-Foxo1 | WB/IF | 1:1000/1:200 | CST 2880 |
| Mouse anti-Ngn3 | IF | 1:100 | DSHB F25A1B3 |
| Mouse anti-NKX6.1 | IF | 1:10 | DSHB F55A10 |
| Mouse anti-mCherry | IF | 1:500 | ABclonal AE002 |
| Rabbit anti-MafA | IF | 1:100 | Abcam ab264418 |
| Alex Fluor 488 Goat Anti-Mouse | IF | 1:200 | Proteintech |
| Alex Fluor 488 Goat Anti-Rabbit | IF | 1:200 | Proteintech |
| Alex Fluor 594 Goat Anti-Mouse | IF | 1:200 | Proteintech |
| Alex Fluor 594 Goat Anti-Rabbit | IF | 1:200 | Proteintech |

CTS, Cell Signaling Technology; DSHB, Developmental Studies Hybridoma Bank; IF, immunofluorescence; WB, western blot;

**Table S2.** The primer sequences of human mRNAs

| Gene | Primer sequences |
| --- | --- |
| β-actin | F: 5′-GGACTCCTATGTGGGTGACG-3′ |
|  | R: 5′-CTTCTCCATGTCGTCCCAGT-3′ |
| Sidt2 | F: 5′-TAGTGCCTGTTACCACGTCTGC-3′ |
|  | R: 5′-GGATGCAGTCTGTGTAGAGCACA-3′ |
| Pdx1 | F: 5′-GCCCGGGTGTAGGCAGTAC-3′ |
|  | R: 5′-CAGTGGGCAGGAGGTGCTTA-3′ |
| MafA | F: 5′-GGAGGTCATCCGACTGAAACA-3′ |
|  | R: 5′-GCACCTCTCGCTCTCCAGAAT-3′ |
| NKX6.1 | F: 5′-ACCTCTGGACCCGAACTCT-3′ |
|  | R: 5′-CCCCGCCACAATTTCTAGGTT-3′ |
| Slc2a2 | F: 5′-TCTTCACGGCTGTCTCTGTG-3′ |
|  | R: 5′-AATCATCCCGGTTAGGAACA-3′ |
| Ngn3 | F: 5′-TCTCAAGCATCTCGCCTCTTC-3′ |
|  | R: 5′-ACAGCAAGGGTACCGATGAGA-3′ |
| MafB | F: 5′-AGGACCTGTACTGGATGGC-3′ |
|  | R: 5′-CACTACGGAAGCCGTCGAAG-3′ |

**Table S3.** The primer sequences of rat mRNAs

| Gene | Primer sequences |
| --- | --- |
| β-Actin | F: 5′-CGAGTACAACCTTCTTGCAGC-3′ |
|  | R: 5′-CAGGTAGGACTGCACCTTCA-3′ |
| Sidt2 | F: 5′-GCAACCTCAGCGCCTTCAACA-3′ |
|  | R: 5′-CGTTGATATCTGGGTGCCGCTTC-3′ |
| Pdx1 | F: 5′-GACCCGTACAGCCTACACTCG-3′ |
|  | R: 5′-CCGCTCGTTGTCCCGCTA-3′ |
| MafA | F: 5′-CAACAGCTGCCCACTAGTCA-3′ |
|  | R: 5′-GCGAAGCTCTGGCCCCGGAA-3′ |
| NKX6.1 | F: 5′-TTCTCCGGCCAGCAGATCTTCGC-3′ |
|  | 5′-TGCCTCCGCTGGATTTGTGCTTT-3′ |
| Slc2a2 | F: 5′-CAACATGTCAGAAGACAAGATCAC-3′ |
|  | R: 5′-CAAGAGGGCTCCAGTCAACG-3′ |
| NKX2.2 | F: 5′-AGCACATGCAATACAACGCC-3′ |
|  | R: 5′-GAAGCGAAGCTGCACAAACA-3′ |


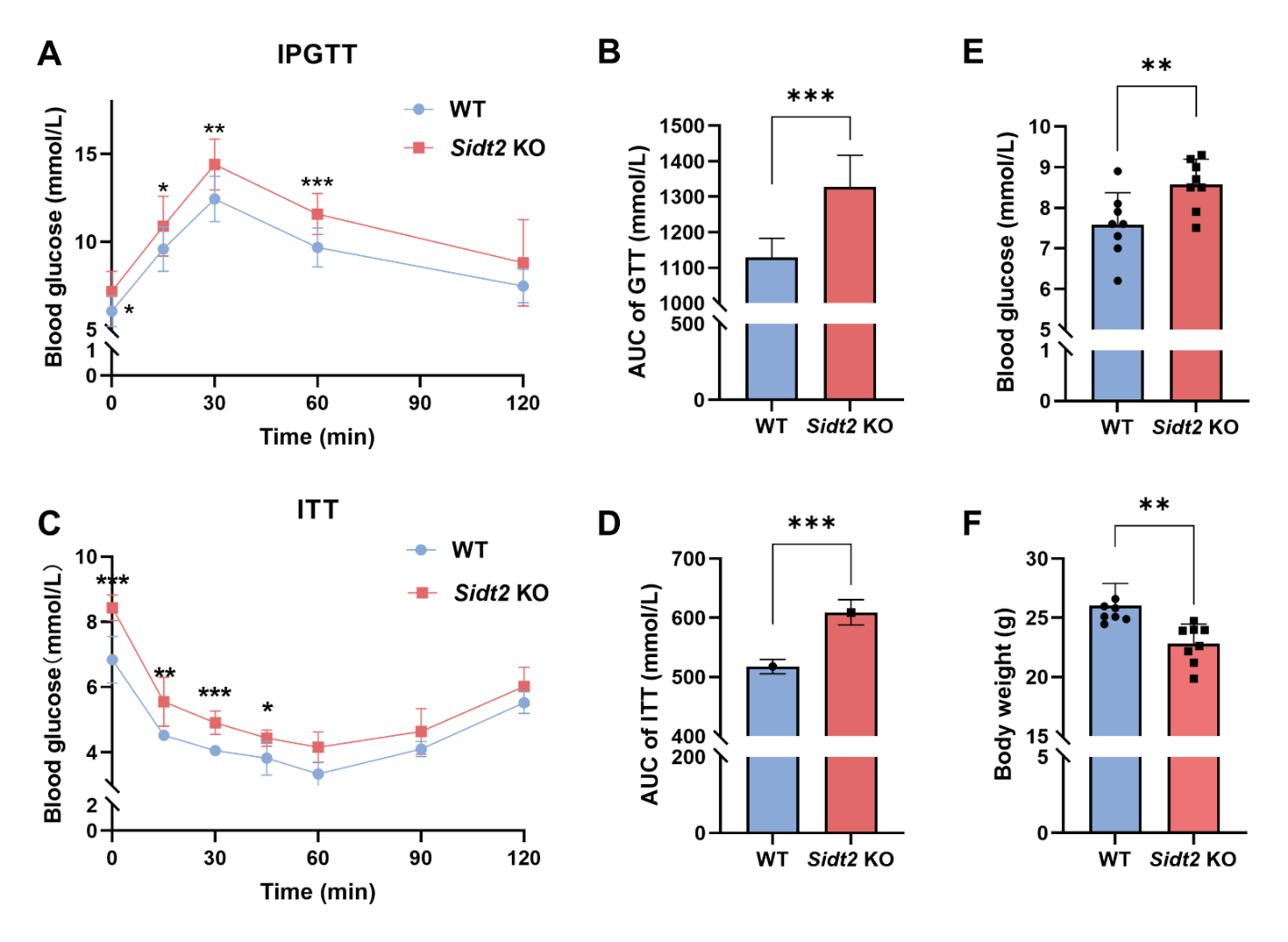


**Figure S1.** Glucose metabolism in *Sidt2* knockout (KO) mice

(A) Glucose tolerance test on 12-week-old wild-type (WT) and *Sidt2* KO mice. (B) Statistical analysis of data from (A). (C) Insulin tolerance test on 12-week-old WT and *Sidt2* KO mice. (D) Statistical analysis of data from (C). (E) Blood glucose levels after fasting for 6 h. (F) Body weight. **P*<0.05, ***P*<0.01, ****P*<0.001.


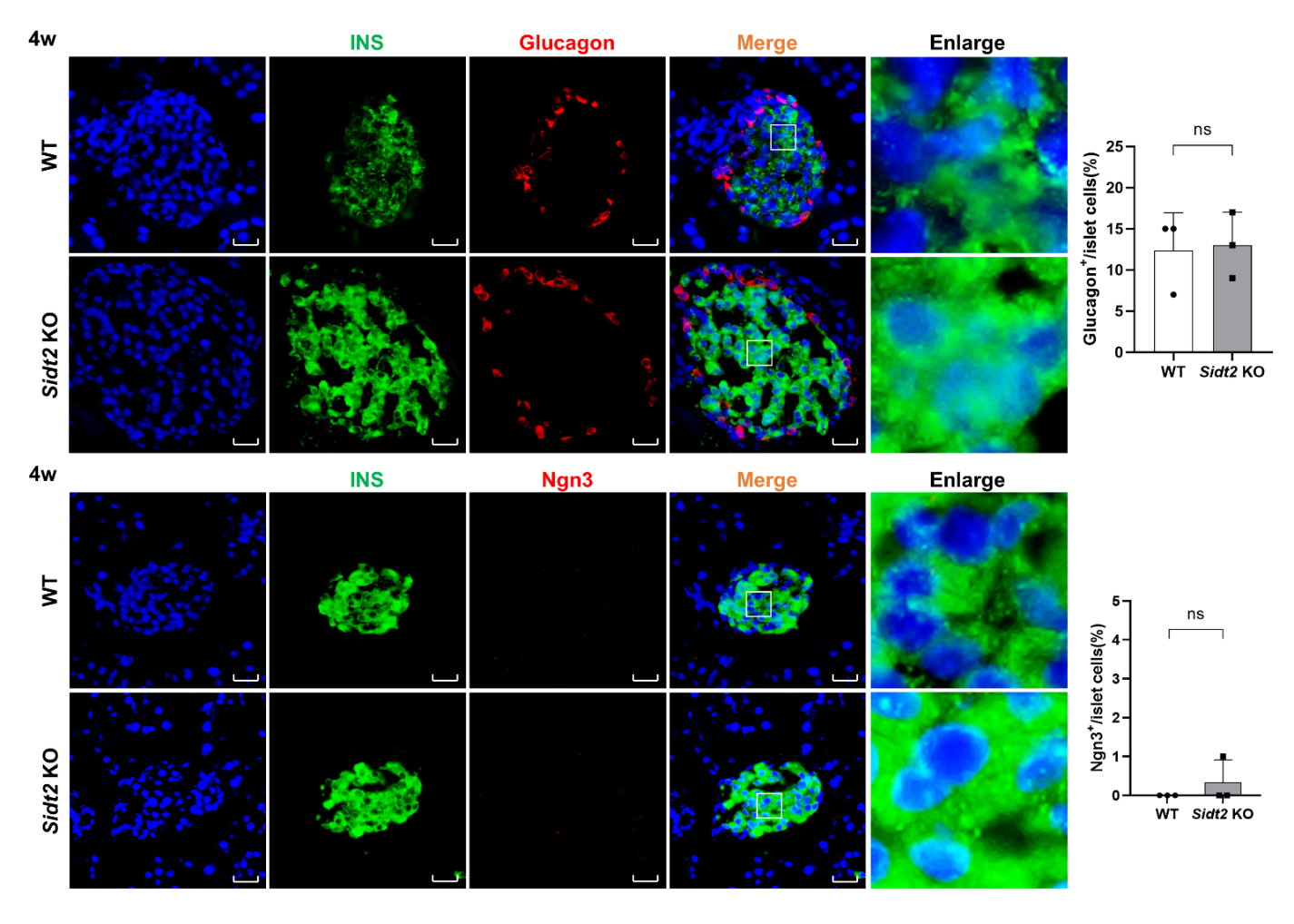


**Figure S2.** Expression of glucagon and Ngn3 in pancreatic islets of one-month-old mice

(A) Immunofluorescence of insulin (green) and glucagon (red) in the pancreas of one-month-old mice. Scale bar=20 μm. (B) Immunofluorescence of insulin (green) and Ngn3 (red) in the pancreas of one-month-old mice. Scale bar=20 μm.


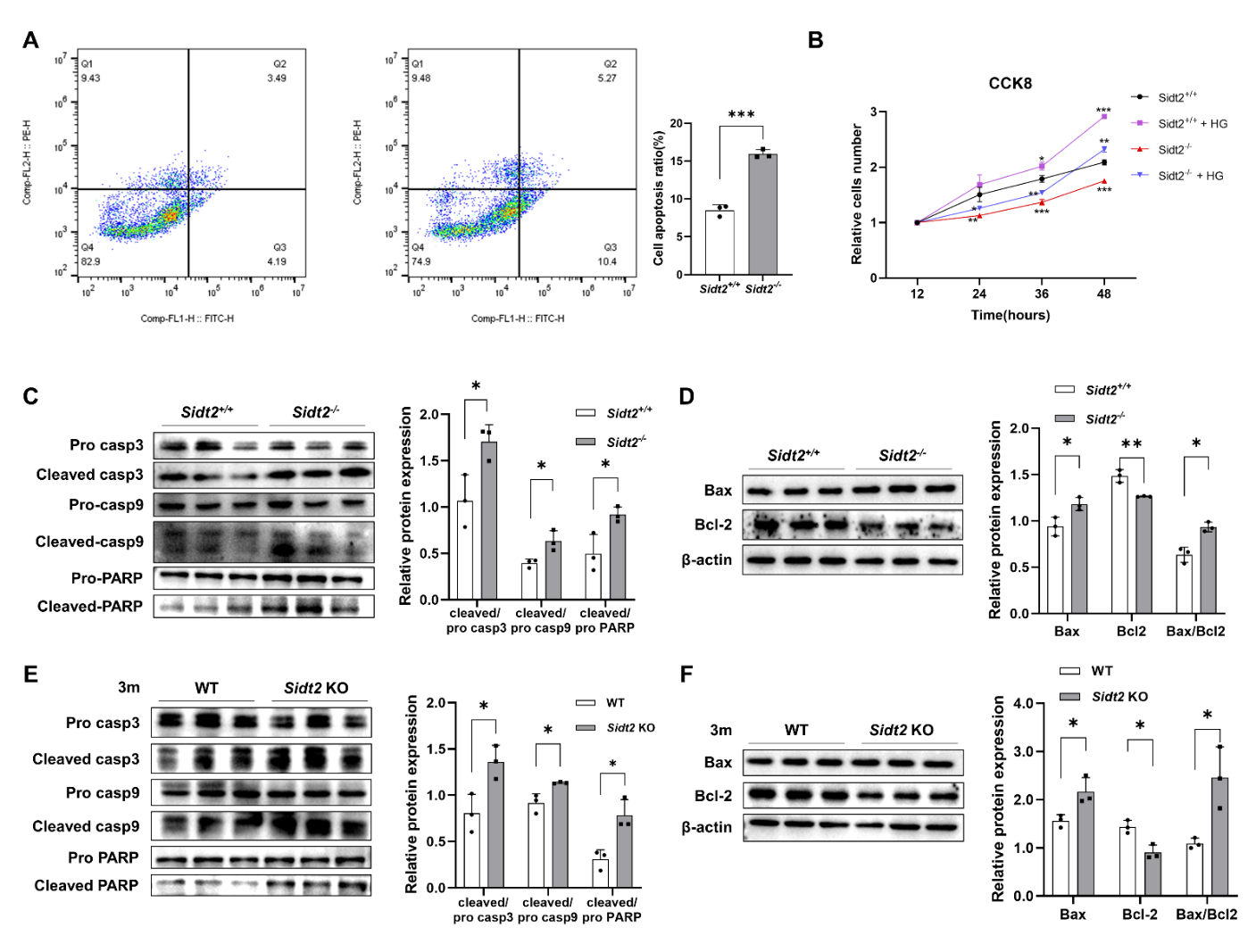


**Figure S3.** Islet survival after *Sidt2* ablation

(A) FITC/PI assay in *Sdit2^+/+^* (left) and *Sidt2^-/-^* (right) INS-1 cells. (B) CCK-8 assay detects the proliferation of INS-1 cells. (C) Expression levels of apoptosis-related proteins in INS-1 cells. (D) Expression levels of Bax and Bcl2 proteins in INS-1 cells. (E) Expression levels of apoptosis-related proteins in islets. (F) Expression levels of Bax and Bcl2 proteins in islets. **P*<0.05, ***P*<0.01, ****P*<0.001.
